# PM_10_ exposure interacts with abdominal obesity to increase blood triglycerides: a cross-sectional linkage study

**DOI:** 10.1093/eurpub/ckab190

**Published:** 2021-11-11

**Authors:** Vânia Gaio, Rita Roquette, Alexandra Monteiro, Joana Ferreira, Diogo Lopes, Carlos Matias Dias, Baltazar Nunes

**Affiliations:** 1 Department of Epidemiology, Instituto Nacional de Saúde Doutor Ricardo Jorge IP (INSA, IP), Lisboa, Portugal; 2 NOVA National School of Public Health, Public Health Research Centre, Universidade NOVA de Lisboa, Lisboa, Portugal; 3 Nova IMS Information Management School, Universidade NOVA de Lisboa, Lisboa, Portugal; 4 CESAM and Department of Environment and Planning, Universidade de Aveiro, Aveiro, Portugal

## Abstract

**Background:**

Blood lipids and glucose levels dysregulation represent potential mechanisms intermediating the adverse cardiovascular effects of ambient particulate matter (PM) exposure. This study aims to estimate the effect of long-term PM_10_ exposure on blood lipids and glucose levels and to assess the potential mediation and/or modification action of abdominal obesity (AO) (waist-to-height ratio).

**Methods:**

Our study was based on 2,390 participants of the first Portuguese Health Examination Survey (INSEF, 2015) with available data on blood lipids and glucose parameters and living within a 30-km radius of an air quality monitoring station with available PM_10_ measurements. PM_10_ concentrations were acquired from the air quality monitoring network of the Portuguese Environment Agency. Generalized linear models were used to assess the effect of 1-year PM_10_ exposure on blood lipids and glucose levels. An interaction term was introduced in the models to test the modification action of AO.

**Results:**

We found an association between PM_10_ and non-fasting blood triglycerides (TG) after adjustment for age, sex, education, occupation, lifestyles-related variables and temperature but only in participants with AO. Per each 1 µg/m^3^ PM_10_ increment, there was a 1.84% (95% confidence interval: 0.02–3.69) increase in TG. For the remaining blood lipid and glucose parameters, no associations were found.

**Conclusions:**

Our study demonstrates that even at low levels of exposure, long-term PM_10_ exposure interacts with AO to increase blood TG. Our findings suggest that reducing both AO prevalence and PM_10_ below current standards would result in additional health benefits for the population.

## Introduction

Ambient particulate matter (PM) exposure is a major global environmental problem and is a recognized factor to develop cardiovascular diseases, the leading cause of death globally.^1^^,^^2^ Blood glucose and lipids levels dysregulation represent potential mechanisms intermediating the cardiovascular adverse effect of the PM exposure. Some epidemiologic studies assessed the association between air pollutants exposure and uncontrolled blood glucose and lipid levels.^3^^,^^4^ However, evidence on this association is still inconsistent.^5^^,^^6^ In the particular case of blood lipid levels, a recent published systematic review and meta-analysis suggests already some epidemiologic evidence supporting the association between PM_10_ (particles with an aerodynamic equivalent diameter ≤10µm) and blood levels of triglycerides (TG). Per each 10 µg/m^3^ PM_10_ increment there was a 3.14% (95% confidence interval [CI]: 1.36–4.95) increase in the TG values. However, only three studies were meta-analysed and more epidemiologic studies are essential to clarify the strength of this association.^5^

PM has been suggested as acting as an environmental endocrine disruptor and one potential biological mechanism explaining its deleterious effect on the blood glucose and lipid levels is through adipokines dysregulation at the adipose tissue level.^7^^,^^8^ Actually, some *in vivo* exposure studies, in animal models, demonstrate that PM exaggerates visceral adipose tissue (VAT) and increase adipokines secretion.^9^^,^^10^ Subsequently, a wide range of physiological mechanisms are induced, including insulin resistance and consequently blood glucose levels increase and also uncontrolled lipolysis, leading to inflated fatty acids delivery to the liver, which will in turn act as subtract to promote lipid synthesis and raised blood lipids levels, mainly TG levels.^11^ Therefore, we hypothesize that VAT could be a mediator of PMs effect on blood glucose and lipid levels because PM will exaggerates VAT and then the subsequent mechanisms will be activated up to dysregulated glucose and lipid metabolism. On the other hand, VAT could also be considered a modifier of the PM effect on blood glucose and lipid levels because the pre-existing VAT could interact with PM to induce adipokines secretion and subsequent uncontrolled blood lipids and glucose levels.

Taking into account the hypothesis previously described, the present study aims (1) to estimate the effect of PM_10_ exposure on blood lipid and glucose levels (TC, total cholesterol; HDL-C, high-density lipoprotein cholesterol; LDL-C, low-density lipoprotein cholesterol; HbA1c, glycated haemoglobin) in the adult Portuguese mainland population and (2) to assess the potential mediation and/or modification action of abdominal obesity (AO) (as a proxy of VAT) on this effect.

## Methods

### Study population

**Figure 1:**
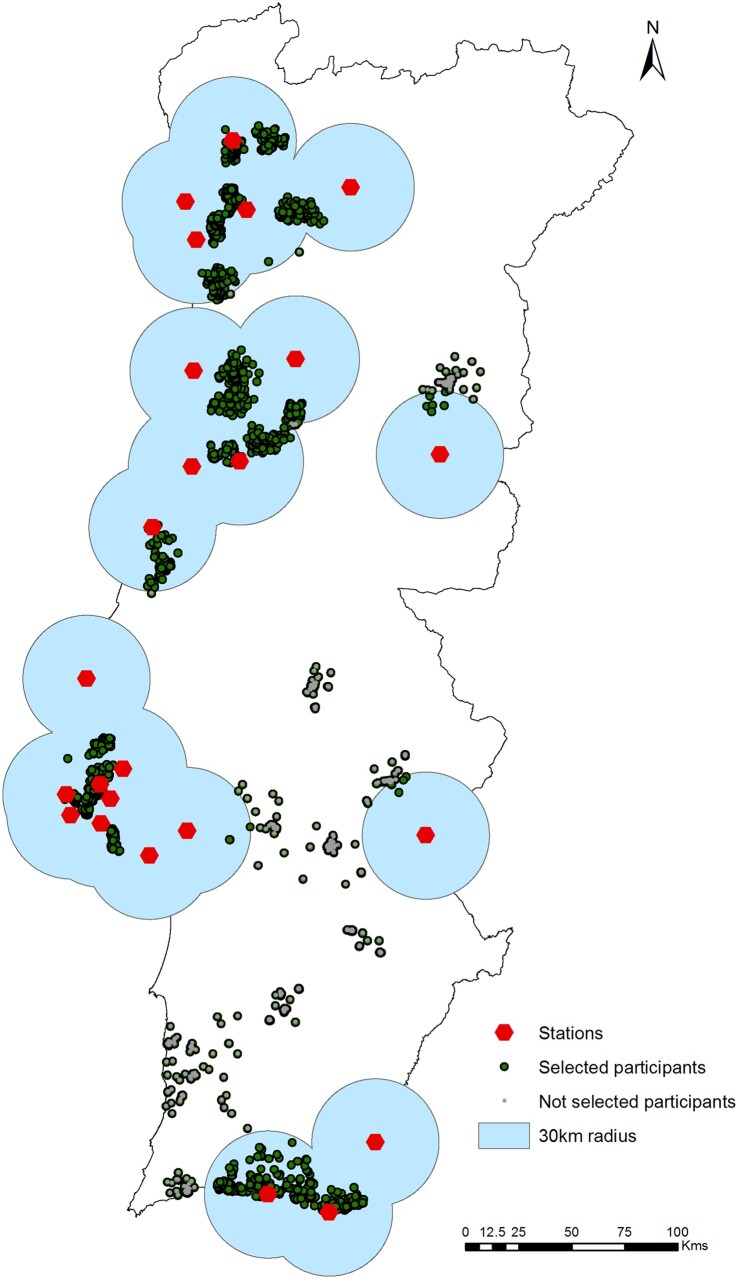
Participant selection flow diagram

This study was conducted using data from the first Portuguese National Health Examination Survey (INSEF), collected between February and December 2015. This survey was described in more detail by Nunes et al.^12^ This analysis was restricted to the subsample of INSEF participants from mainland Portugal (*n* = 3,467) with participants consent to link data, available data on zip code number, living within a 30-km radius of an air quality monitoring station with available PM_10_ concentration values and available data on blood lipids or glucose parameters (*n* = 2,390) (figure 1).

The INSEF survey received approval from the Ethics Committee of the Portuguese National Health Institute Doutor Ricardo Jorge, the National Data Protection Authority (Authorization no. 9348/2010) and from the regional Ethics Committees.

### Health data

Health data collection was performed by trained health professionals, according to the European Health Examination Survey (EHES) procedures.^13^ HbA1C was measured in fresh non-fasting whole blood samples and blood lipids (TC, HDL, LDL and TG) were measured in fresh non-fasting serum samples, in the 12 regional collaborating laboratories that participated in the National Program for External Quality Assessment (PNAEQ) to assure comparability and reliability of blood tests results.

Waist-to-height ratio (WHtR) was used as proxy of AO because in the absence of more objective measures of central obesity and adiposity, it is the most suitable proxy measure of the VAT quantity.^14^ WHtR was assessed using waist circumference and height measurements, assuming that participants with a WHtR ≥ 0.5 had AO.

Sociodemographic (age, sex, educational level and occupation), lifestyle (smoking, excessive alcohol consumption, sedentary and unhealthy diet) and health status variables (diagnosed-dyslipidaemia, diagnosed diabetes, lipid-lowering medication usage and diabetes medication usage) were obtained by self-report through the interview.

Regarding educational level, we considered the highest level of education completed, grouped into three categories, according the 2011 International Standard Classification of Education (ISCED)^15^: low education (levels 0–2 of the ISCED 2011), medium education (levels 3–4 of the ISCED 2011) and high education (levels 5–8 of the ISCED 2011). Occupation was grouped according to the International Standard Classification of Occupations (ISCO-08)^16^ into two categories: white-collar occupation (Managers, Professionals, Technicians and Associate Professional, Clerical Support Workers and Services and Sales Workers) and blue-collar occupation (Skilled Agricultural Workers, Craft and Related trades Workers, Plant and Machine Operators and Elementary occupations).

Regarding the lifestyles-related variables, smokers, excessive alcohol consumption, unhealthy diet and sedentary were defined as previously reported.^17^

### Environmental exposure assessment

**Figure 2:**
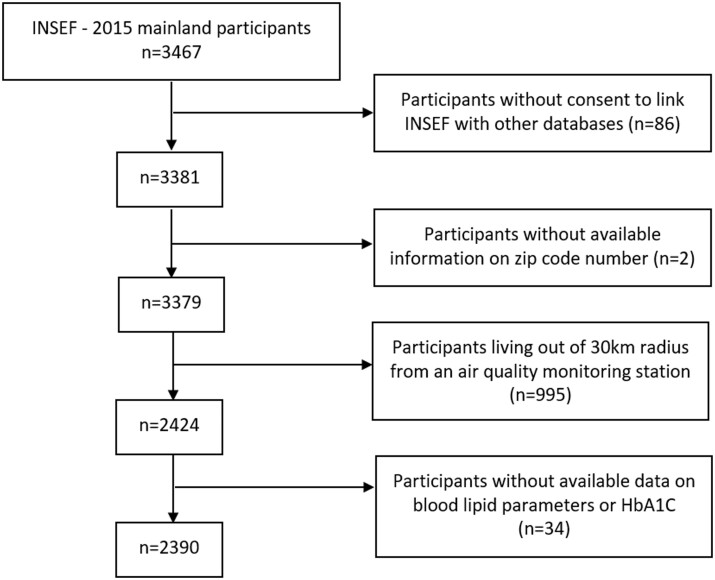
Geographic distribution of the participants and the 24 background air quality monitoring stations with available PM_10_ data during the study period. Red points represent the air quality monitoring stations, green points represent the INSEF Portuguese mainland participants and blue circles represent the 30-km radius from each station. The grey points not covered by the blue circles are the excluded participants. (Names of the air quality monitoring stations: Alverca, Arcos, Cerro, Douro Norte, Ervedeira, Fernando Pó, Fornelo do Monte, Frossos-Braga, Fundão, Ílhavo, Instituto Geofísico de Coimbra, Joaquim Magalhães, Laranjeiro, Loures-Centro, Lourinhã, Malpique, Mem Martins, Mindelo-Vila do Conde, Montemor-o-Velho, Olivais, Paços de Ferreira, Quinta do Marquês, Sobreiras-Lordelo do Ouro and Terena).

We obtained PM_10_ values from QualAr database, available online at the Portuguese Environment Agency (APA) website (https://qualar.apambiente.pt/). We assumed the period of 1-year PM_10_ exposure as being representative of participants long-term PM_10_ exposure as they reported to live in the same place at least 1 year before the INSEF examination day. Only background stations with data collection efficiency of at least 75% were considered. The geographic distribution of the participants (zip code number) and the 24 background air quality monitoring stations are shown in figure 2. Daily average PM_10_ concentrations were calculated, in all INSEF fieldwork days, using the 24-h observations values from each station. One-year average PM_10_ concentrations were estimated using the preceding 365-daily average PM_10_ concentrations values. For each individual, the allocated 1-year average PM_10_ concentration was the weighted average of 1-year averages PM_10_ concentrations of all stations within 30 km from that participant’s. This average was weighted by the inverse of the squared distance between the residence and the air quality monitoring stations, as previously reported.^17^

For each individual, the allocated 1-year average temperatures were obtained using data from the National Oceanic and Atmospheric Administration database (www.ncdc.noaa.gov) and we assumed the 1-year average value of the closest temperature monitoring station as being representative of the individual exposure.

### Statistical analysis

The statistical analysis was performed using the R program (version 3.6.3).^18^ The significance level for all analysis was set at 5%. Sampling weights were used in data analysis. All estimates were weighted to account for different selection probabilities resulting from complex sample design and to match the population distribution in terms of geographic region, age group and sex, in 2015. *T*-test and the Wilcoxon test were used to access differences of quantitative variables according to their adherence to the normal distribution or not. Proportions were compared using Pearson’s Chi-squared test.

### Conceptual model

We constructed a directed acyclic graph (DAG) shown in Supplementary figure S1 based on literature review to select the minimal sufficient adjustment set of variables needed to account for confounding of the exposure–outcome relationship. This analysis was performed using the ‘DAGitty’ R package.^19^

### Primary analysis

Regression coefficients of effect (*β*) of PM_10_ on TC, TG, LDL-C, HDL-C and HbA1C with the corresponding 95% CIs were obtained by generalized linear regression models analyses for each 1-μg/m^3^ increment of PM_10_. Then, percent change with corresponding 95% CIs were calculated by using the formula 100 × [exp (*β*)−1]. We used the svyglm function from the ‘survey’ R package to run each Gaussian family model with a link function log (family= gaussian(link = ‘log’)).

First, an unadjusted exposure–outcome model was fitted for each outcome. Then, a second model confounder-adjusted for sex (male/female), age group (50 years; ≥50 years), educational level (low education/medium education/high education), occupation (white-collar occupation/blue-collar occupation), smoking (smoker/no smoker), excessive alcohol consumption (yes/no), sedentary (yes/no), unhealthy diet (yes/no) and individual allocated 1-year average temperature (continuous) was performed for each outcome.

To determine whether AO is a potential mediator between PM_10_ exposure and parameters levels, we performed a mediation analysis according to Jonhson et al.^20^ To determine if AO interact with PM_10_ levels, an interaction term (PM_10_*AO) was introduced in the final model of each outcome.^21^ If the *P* values were statistically significant (*P* < 0.05), an AO-stratified analysis was performed.

### Sensitivity analysis

To assess the sensitivity of our analysis to the 30-km radius criteria, we also fit the models for each outcome considering only participants living within a 20-km radius of an air quality monitoring station with available PM_10_ measurements.

Additionally, to evaluate the sensitivity of our analysis regarding the exposure assessment method, we also fit the models considering the modelled PM_10_ concentrations obtained by the application of an air quality modelling system composed by the Weather Research & Forecasting (WRF, version 3.7.1)^22^ and Comprehensive Air Quality Model with Extensions (CAMx, version 6.40).^23^ The WRF-CAMx system has been extensively applied for Portugal and worldwide and it is described in more detail elsewhere.^24–26^ It returns surface hourly average concentrations of simulated species by grid cell (5 × 5 km^2^) that were used to compute PM_10_ daily averages in 2014 and 2015. Participants living within a 30-km radius of at least one air quality monitoring station were linked to the correspondent grid cell and grid cell’s PM_10_ daily averages were considered as being representative of the individual exposure. The preceding 365-daily average PM_10_ concentrations at the INSEF examination day were considered to obtain the individual allocated 1-year average PM_10_ concentrations.

To assess the sensitivity of our analysis to the choice of AO assessed by the WHtR as the VAT proxy, we also use waist-to-hip ratio as a proxy of VAT and repeat the stratified analysis.

Finally, we also repeat the primary analysis after excluding the participants with diagnosed dyslipidaemia or taking lipid-lowering medication (in the TG, CT, HDL-C and LDL-C models) and diabetic participants or taking medication for diabetes treatment (in the HbA1C model).

## Results

### General characteristics of participants

Included and excluded participants were similar regarding the majority of the analysed characteristics. Differences between the two groups were only found regarding the percentage of smokers, prevalence of diagnosed diabetes and medicated diabetic participant’s percentage (Supplementary table S1).

Among the 2,390 participants in our study, 52.59% were females, 52.76% aged between 25 and 49 years old, 58.44% had low education level and 62.61% had a white-collar occupation. Most participants reported to be non-smokers (79.05%), non-excessive alcohol consumers (63.93%), to have a healthy diet (63.90%) and to be not sedentary (57.47%). The prevalence of diagnosed-dyslipidaemia and diagnosed-diabetes was 24.95% and 7.78%, respectively, and 19.34% of the participants reported to take lipid-lowering medication and 7.09% reported to take diabetes medication. Individual allocated 1-year average temperature was 15.7°C and individual allocated 1-year average PM_10_ concentration was 17.6 µg/m^3^ (table 1).

**Table 1:** General characteristics of the study participants, according to their AO condition

Characteristics	Participants with AO (*n* = 1,831)	Participants Without AO (*n* = 536)	Total participants (*n* = 2,390)
Sex (*n* = 2,390) (%)			
Males	48.80	42.84	47.41
Females	51.20	57.15	52.59
**Age (*n* = 2,390) (%)**			
**25–49 years old**	**42.66**	**82.96**	**52.76**
**50–74 years old**	**57.34**	**17.04**	**47.24**
**^a^Level of education (*n* = 2,389) (%)**			
**Low education**	**66.37**	**35.07**	**58.44**
**Medium education**	**19.00**	**29.38**	**21.86**
**High education**	**14.63**	**35.55**	**19.70**
**^b^Occupation (*n* = 2,203) (%)**			
**White-collar occupation**	**57.98**	**76.18**	**62.61**
**Blue-collar occupation**	**42.02**	**23.82**	**37.39**
**Lifestyles variables (%)**			
**^c^Smokers (*n* = 2,390)**	**17.44**	**32.22**	**20.95**
**^d^Excessive alcohol consumers (*n* = 2,388)**	**40.21**	**24.49**	**36.07**
**^e^Unhealthy diet (*n* = 2,388)**	**33.69**	**43.36**	**36.10**
**^f^Sedentary (*n* = 2,375)**	**46.01**	**39.15**	**44.53**
**Diagnosed dyslipidaemia (*n* = 2,373) (%)**	**30.64**	**8.26**	**24.95**
**Dyslipidaemia medication (*n* = 2,390) (%)**	**24.33**	**4.45**	**19.33**
**Diagnosed diabetes (*n* = 2,384) (%)**	**9.99**	**0.10**	**7.78**
**Diabetes medication (*n* = 2,390) (%)**	**9.12**	**0.10**	**7.09**
Individual allocated 1-year average temperature (*n* = 2,390) (**°**C) (mean±SD)	15.58 ± 1.42	15.90 ± 1.44	15.66 ± 1.43
Individual allocated 1-year average PM_10_ (*n* = 2,390) (µg/m^3^) (mean±SD)	17.54 ± 3.01	17.87 ± 2.74	17.63 ± 2.95
**Outcome variables**			
**TC (*n* = 2,390) (mg/dL)** (mean±SD)	**196.45 ± 37.50**	**184.25 ± 34.98**	**193.65 ± 37.74**
**HDL-C (*n* = 2,390) (mg/dL)** (mean±SD)	**52.04 ± 13.16**	**59.82 ± 14.54**	**54.03 ± 14.02**
**LDL-C (*n* = 2,390) (mg/dL)** (mean±SD)	**131.73 ± 34.14**	**116.94 ± 31.70**	**128.18 ± 34.39**
**TG (*n* = 2,390) (mg/dL)** (mean±SD)	**162.63 ± 102.61**	**99.87 ± 56.25**	**147.35 ± 97.36**
**HbA1C (*n* = 2,357) (%)** (mean±SD)	**5.55 ± 0.76**	**5.17 ± 0.45**	**5.45 ± 0.72**

Results in bold are those with statistically significant difference between participants with versus without AO, according to the Pearson’s Chi-squared test (*P *<* *0.05).

a Low education: levels 0–2 of the ISCED 2011^15^; medium education: levels 3–4 of the ISCED 2011,^15^ high education: levels 5–8 of the ISCED 2011.^15^

b White-collar occupation: Managers, Professionals, Technicians and Associate Professional, Clerical Support Workers and Services and Sales Workers^16^; blue-collar occupation: Skilled Agricultural Workers, Craft and Related trades Workers, Plant and Machine Operators and Elementary occupations.^16^

c Smokers include current daily and occasional smokers.

d Three or more days/week of consumption of at least one of the following alcoholic beverages (wine, beer, brandy/bagasse, port wine/Martini/liqueur, whisky/gin/vodka).

e No consumption of fruit and vegetables at least once a day.

f Reading, watching TV or other sedentary activities declared as the best description of the leisure time activities during the last 12 months.

The individual allocated 1-year average PM_10_ concentration values ranged between 10.45 and 26.16 µg/m^3^ (median = 18.51 µg/m^3^, interquartile range [IQR] = 15.27–19.28 µg/m^3^). The mean concentrations of TC, TG, HDL-C and LDL-C were 193.65, 147.35, 54.03 and 128.18 mg/dL, respectively. The mean percentage of HbA1C was 5.45%. When comparing participants with and without AO, differences were found regarding age, level of education, occupation, lifestyles-related variables, diagnosed dyslipidaemia and diabetes, medicated participants and outcome variables (table 1).

### Primary analysis

There was an association between PM_10_ and blood TG levels after adjustment for age, sex, educational level, occupation, variables, lifestyles and annual mean temperatures. Per each 1 µg/m^3^ PM_10_ increment there was a 1.70% (95% CI: 0.11–3.32) increase in the TG values of the participants. No associations were found for the remaining blood lipid parameters and HbA1C (table 2).

**Table 2:** Percent changes in TG, TC, HDL-C, LDL-C and HbA1C per 1 µg/m^3^ increment of PM_10_ among all participants, participants with AO and participants without AO

	% Change per 1 µg/m^3^ of PM_10_ increment				
	TG	TC	HDL-C	LDL-C	HbA1C
**All included participants (*n* = 2,390)**					
Not adjusted model	0.19	0.09	0.12	−0.89	−0.09
	(−1.08; 1.48)	(−0.52; 0.70)	(−0.43; 0.68)	(−1.87; 0.11)	(−0.37; 0.19)
^a^Adjusted model	**1.70**	0.59	−0.20	0.47	−0.01
	**(0.11; 3.32)**	(−0.07; 1.24)	(−0.74; 0.33)	(−0.20; 1.15)	(−0.48; 0.47)
**Participants with AO (*n* = 1,831)** ^b^					
Not adjusted model	0.92	1.75	−0.21	−0.64	−0.09
	(−0.60; 2.46)	(−0.45; 0.80)	(−0.69; 0.27)	(−1.66; 0.39)	(−0.45; 0.28)
^a^Adjusted model	**1.84**	0.62	−0.38	0.56	−0.03
	**(0.02; 3.69)**	(−0.02; 1.27)	(−0.97; 0.21)	(−0.22; 1.35)	(−0.62; 0.57)
**Participants without AO (*n* = 536)**					
Not adjusted model	−1.76	0.03	0.64	−**1.32**	0.20
	(−3.60; 0.12)	(−0.71; 0.78)	(−0.37; 1.66)	**(**−**2.39;** −**0.24)**	(−0.19; 0.60)
^a^Adjusted model	0.75	0.62	0.46	0.23	0.07
	(−1.90; 3.47)	(−0.38; 1.63)	(−0.52; 1.45)	(−0.66; 1.12)	(−0.45; 0.60)

Results in bold are those statistically (*p*<0.05).

a Adjusted for age, sex, educational level, occupation, smoking status, excessive alcohol consumption, unhealthy diet, sedentary and individual allocated 1-year average temperature.

b Participants without available data on waist or height measurements (*n* = 23) and consequently without AO data were excluded from the stratified analysis.

We detected an interaction between PM_10_ and AO in the TG analyses (interaction term: 1.024, 95% CI: 1.002–1.046, *P*-values: 0.034), and, consequently, we present a stratified analysis in table 2. As we can see, the association between PM_10_ and TG levels was only found in participants with AO. Per each 1 µg/m^3^ PM_10_ increment, there was a 1.84% (95% CI: 0.02–3.69) increase in the TG values of the participants with AO (table 2).

We found that there was no association between the exposure (PM_10_) and the mediator (AO), a condition required to perform the mediation analysis. Consequently, the mediation analysis could not be done and we assumed that, based on our results, there was no evidence to suggest that AO mediate the association between PM_10_ and blood lipid or glucose levels.

### Sensitivity analysis

When we restricted our sample to the participants living within a 20-km radius of an air quality monitoring station with available PM_10_ values, similar results to those from the primary analysis were found (Supplementary table S2). Additionally, we found an association between PM_10_ and blood CT levels after adjustment for confounding in the all participant’s analysis and also in the participants with AO. Per each 1 µg/m^3^ PM_10_ increment there was a 0.53% (95% CI: 0.11–0.94) increase in the TG values of the participants with AO (Supplementary table S2).

When we considered the individual allocated 1-year average PM_10_ concentrations obtained by the air quality modelling system (WRF-CAMx), no associations were found (Supplementary table S3). We obtained similar results to those from the primary analysis when we excluded participants with diagnosed dyslipidaemia or taking lipid-lowering medication (in the TG, CT, HDL-C and LDL-C modelling) and diabetic participants or taking medication for diabetes treatment (in the HbA1C modelling) (Supplementary table S4). The changing of the AO measure from WHtR to waist-to-hip ratio also did not modify the obtained results (Supplementary table S5).

## Discussion

### Key findings

Our results showed that at least 1-year PM_10_ exposure interacts with AO to increase non-fasting blood TG levels by about 2% per each 1 µg/m^3^ PM_10_ increase, in individuals with AO. For the remaining blood lipid and glucose parameters, no associations were found.

We were able to detect the effect modification of AO (as a proxy of VAT) and, as hypothesized, the biological mechanism explaining our results could be the interaction between the pre-existing quantity of VAT with PM_10_ that will induce adipokines secretion and subsequent raised blood TG levels.^11^ On the other hand, we also hypothesized the potential mediation action of AO but it was not supported by our results.

All sensitivity analysis strengthens our results except when considering modelled PM_10_ concentrations obtained by an air quality modelling system (WRF-CAMx).

### Comparison with other published studies and interpretations

Our results are in concordance with a recent published meta-analysis that reported some epidemiologic evidence supporting the association between PM_10_ and increased blood TG levels.^5^ However, we detected this association only in participants with AO, contrary to the previously reported studies.^27–29^ Moreover, our estimate was much higher but less precise than the one reported by Cai et al.^28^ that studied two large European cohorts exposed to a similar levels of PM_10_ concentrations (1.70% blood TG increase per 1 µg/m^3^ PM_10_ increment [95% CI: 0.11–3.32] versus 1.9% blood TG increase per 2 µg/m^3^ PM_10_ increment [95% CI: 1.5–2.4]). Precision differences are probably due to the huge sample size differences (*n* = 2,390 versus *n* = 111,547) and the estimate magnitude difference can be explained by the different set of adjustment variables.

Taking into account the biological mechanism hypothesized, it makes sense that TG levels are the most sensitive blood lipid parameters because additional adipokine secretion, namely TNF-alpha, will induce uncontrolled fatty acid lipolysis from VAT, leading to inflated fatty acids delivery to the liver, which will act as subtract to promote mainly TG synthesis.^11^ Moreover, the loss of insulin sensitivity within adipose tissue induced by additional adipokine secretion could be not directly reflected on the blood HbA1C levels, explaining why we did not found an effect of PM_10_ on this blood parameter.^11^

Despite our study being the first to report the modification effect of the AO regarding the PM_10_ effect on blood lipid levels, it had been recently reported regarding the air pollution effect on other health outcomes, namely blood pressure,^30^ kidney function^31^ and lung function.^32^ Moreover, previous studies performed in China and USA, already reported stronger associations between long-term PM exposure and blood lipids levels in overweight or obese participants.^27^^,^^33^

Regarding the sensitivity analysis, it strengthen our results, suggesting that estimates from the primary analysis could be underestimated due to the exposure misclassification and outcome misclassification associated to the inclusion of participants with dyslipidemia or taking lipid lowering medication. When considering modelled PM_10_ concentrations obtained by an air quality modelling system (WRF-CAMx) no statistically significant results were found. These modelled data were obtained using a numerical air quality modelling system (WRF-CAMx) with good performance already reported for Portugal domain applications.^23^^,^^25^ Nevertheless, modelled PM_10_ concentrations, when compared with measured PM_10_ concentrations, could be less representative of the real participants PM_10_ exposure because they are a mathematical representation of the reality with a certain degree of uncertainty of the input data, namely in the atmospheric emissions data.

### Strengths and limitations

This is the first Portuguese study that links health data from a National Health Examination Survey and air quality monitoring data. We have done an extensive bibliographic review and construct a conceptual model previous to the statistical analysis, in order to guarantee that the main confounding variables on the relationship between PM_10_ and blood lipid and glucose values were considered. Moreover, we consider multiple methodologies to obtain PM_10_ concentrations, by both measurements and numerical modelling (WRF-CAMx) approaches, as presented in the sensitivity analysis.

One of the limitations of our study is related to the exposure assessment method, namely the criteria used to select the participants taking into account the distance between their residence and the air quality monitoring stations. In fact, as the sensitivity analyses indicate, we may not be detecting PM_10_ effect on other parameters than TG due to an exposure misclassification. However, the number of air quality monitoring stations in the Portuguese mainland and their spatial distribution does not allow us to apply a more restrictive. Moreover, both measured and modelled PM_10_ concentrations are still problematic regarding their capacity to assess the real individual exposure that have unique activity patterns and only with the use of new technology, with Global Position Systems (GPS) and mobile devices with low-cost air pollution sensors, we could better assess real individualized exposures and reduce exposure misclassification.^34^

Another limitation of our study is related to the size of the PM analysed. It is known that the smaller particles are those penetrating in deep into the lungs having the ability to be translocated into the bloodstream,^35^ potentially being the major contributors to the endocrine disruption at the adipose tissue level. However, we analysed only PM_10_ levels, the ones available to be analysed in our period of study, and we assumed that they are correlated with smaller particles concentrations, as previously reported.^36^

The non-fasting state of the participants could also be considered a limitation of our study, as TG show significant postprandial elevations according to the diet content.^37^ However, in the previous meta-analysis performed, the effect of PM_10_ exposure on TG levels remains even when considering only participants in fasting state.^5^ Additionally, TG in a non-fasting condition could be more informative as it has been shown to be superior to fasting in predicting cardiovascular risk.^38^ Finally, despite we performed the adjustment for several potential confounders, there is still a possibility of residual confounding. Moreover, effect estimates were based on a single-pollutant model and interactions with gaseous pollutants were not evaluated. It is known that there are important interactions between the atmospheric pollutants, namely the potential additive effects of multiple pollutants and they should be considered in future studies.^39^ In the future, it would also be important to test the hypothetical mechanism in this study, *in vitro* or even *in vivo* assays, in order to verify whether the hypothesized biological mechanism could explain the PM effect on blood lipid and glucose levels.

## Conclusions

To the best of our knowledge, this is the first study showing the modification action of AO regarding the PM_10_ effect on a blood lipid parameter (TG). In comparison with other countries in the world, Portugal presents, in the time period under analysis (2014–2015), relatively low values of PM_10_ (range: 10.45–26.16 µg/m^3^) not exceeding the annual limit value imposed by the European Air Quality Standards (annual mean: 40 µg/m^3^). Even so, it was possible to detect the effect of exposure to PM_10_ on the values of one of the lipid parameters. This supports the statement that, as already some authors argue,^40^ there is no safe level of air pollutants, with effects on human health occurring even when air pollutants levels meeting the standards. On the other hand, our study also strongly suggests that the presence of comorbidities such as AO, which affects the majority of the population, not only Portuguese but also worldwide, leaves the population more vulnerable to the effect of air pollutants.

Finally, our study strongly suggests that even at low levels of exposure, PM_10_ interacts with AO to increase blood TG levels and our findings suggest that reducing both AO prevalence and ambient air pollution below current standards would result in additional health benefits for the Portuguese population.

## Supplementary Material

ckab190_Supplementary_Data
