# Supplementary material for: PM10 exposure interacts with abdominal obesity to increase blood triglycerides: a cross-sectional linkage study
Source: Eur J Public Health. 2021 Nov 11;32(2):281–8. doi: 10.1093/eurpub/ckab190 (PMC9090274; doi:10.1093/eurpub/ckab190)
Supplement: ckab190_Supplementary_Data [file ckab190_supplementary_data.docx]

**Table S1.** Comparison of the general characteristics of the included *versus* excluded INSEF mainland participants.

| **Characteristics** | **Included participants**  **(n=2390)** | **Excluded participants**  **(n=1077)** | **Total mainland INSEF participants (n=3467)** |
| --- | --- | --- | --- |
| Sex (n=3467) - % |  |  |  |
| Males  Females | 47.41  52.59 | 47.92  52.08 | 47.50  52.50 |
| Age group (n=3467) - % |  |  |  |
| 25-49 years  50-74 years | 52.76  47.24 | 51.47  48.53 | 52.53  47.47 |
| ^a^ Level of Education (n=3464) - % |  |  |  |
| Low education  Medium education  High education | 58.44  21.86  19.70 | 60.85  20.20  18.95 | 58.86  21.57  19.57 |
| ^b^ Occupation (n=3200) - %  White-collar occupation | 62.61 | 60.13 | 62.18 |
| Blue-collar occupation | 37.39 | 39.87 | 37.82 |
| Lifestyles variables - % |  |  |  |
| **^c^ Smokers (n=3465)**  ^d^ Excessive alcohol consumption (n=3465)  ^e^ Unhealthy Diet (n=3463)  ^f^ Sedentary (n=3443) | **20.95**  36.07  36.10  44.53 | **26.21**  36.00  36.58  45.68 | **21.87**  36.06  36.19  44.73 |
| ^g^ Abdominal Obesity (n=3435) - % | 75.21 | 78.60 | 75.81 |
| Diagnosed Dyslipidaemia (n=3433) - % | 24.95 | 26.74 | 25.26 |
| Lipid-Lowering medication (n=3467) - % | 19.34 | 19.92 | 19.44 |
| **Diagnosed Diabetes (n=3453) - %** | **7.78** | **10.75** | **8.31** |
| **Diabetes medication (n=3467) - %** | **7.09** | **9.87** | **7.58** |
| Individual allocated 1-year average temperature (n=3465) - ºC(mean ± standard deviation) | 15.70±1.4 | 15.30±1.4 | 15.60±1.4 |

**^a^** Low education: levels 0-2 of the ISCED 2011; Medium education: levels 3-4 of the ISCED 2011, High education: levels 5-8 of the ISCED 2011;

**^b^** White-collar occupation: Managers, Professionals, Technicians & Associate Professional, Clerical Support Workers and Services & Sales Workers; Blue–collar occupation: Skilled Agricultural Workers, Craft & Related trades Workers, Plant & Machine Operators and Elementary occupations.

**^c^** Smokers include current daily and occasional smokers.

**^d^** 3 or more days/week of consumption of at least one of the following alcoholic beverages (wine, beer, brandy/bagasse, Port wine/Martini/liqueur, Whisky/Gin/Vodka).

**^e^** No consumption of fruit and vegetables at least once a day.

**^f^** Reading, watching TV or other sedentary activities declared as the best description of leisure time activities during the last 12 months.

**^g^** Waist-to-height ratio (WHtR) ≥0.5.

Results in bold are those with statistically significant difference between included versus excluded INSEF mainland participants, according to the Pearson's chi-squared test *(p*<0.05).

**Table S2-** Percent changes in TG, TC, HDL-C, LDL-C and HbA1C per 1 µg/m^3^ increment of PM_10_ among all participants, participants with AO and participants without AO, assuming the criteria of 20 km instead of 30 km to select the air monitoring stations contributing to Individual allocated 1-year average PM_10_ concentrations.

|  | **% Change per 1 µg/m^3^ of PM_10_ increment** | | | | |
| --- | --- | --- | --- | --- | --- |
|  | TG | TC | HDL-C | LDL-C | HbA1C |
| **All included participants (n=1819)** | | | | | |
| Not adjusted model | 0.27  (-1.00; 1.56) | 0.08  (-0.39; 0.49) | 0.09  (-0.51; 0.69) | -**1.42**  **(-2.47; -0.35)** | 0.02  (-0.22; 0.27) |
| *Adjusted model | **1.78**  **(0.31; 3.28)** | **0.50**  **(0.04; 0.96)** | -0.33  (-1.12; 0.46) | -0.12  (-0.83; 0.60) | 0.17  (-0.13; 0.46) |
| **Participants with AO (n=1363)^a^** |  | | | | |
| Not adjusted model | 0.97  (-0.71; 2.69) | 0.12  (-0.30; 0.53) | -0.22  (-0.78; 0.35) | **-1.30**  **(-2.37; -0.21)** | -0.01  (-0.29; 0.28) |
| *Adjusted model | **2.00**  **(0.26; 3.76)** | **0.53**  **(0.11; 0.94)** | -0.60  (-1.26; 0.07) | -0.06  (-0.82; 0.71) | 0.13  (-0.23; 0.50) |
| **Participants without AO (n=439)** |  | | | | |
| Not adjusted model | -1.59  (-4.18; 1.08) | 0.06  (-0.82; 0.95) | 0.47  (-0.74; 1.69) | **-1.57**  **(-3.04; -0.08)** | **0.33**  **(0.02; 0.65)** |
| *Adjusted model | -0.12  (-3.27; 3.13) | 0.60  (-0.43; 1.64) | 0.40  (-1.14; 1.97) | -0.17  (-1.41; 1.08) | 0.30  (-0.04; 0.64) |

*adjusted for age, sex, educational level, occupation, smoking status, excessive alcohol consumption, unhealthy diet, sedentary and Individual allocated estimated 1-year average temperature.

^a^ Participants without available data on waist or height measurements (n=17) and consequently without AO data were excluded from the stratified analysis.

Abbreviations: AO- Abdominal Obesity; TC - Total Cholesterol; HDL-C – High density Lipoprotein cholesterol; LDL-C – Low density lipoprotein cholesterol; TG – Triglycerides.; PM- Particulate matter.

**Table S3-** Percent changes in TG, TC, HDL-C, LDL-C and HbA1C per 1 µg/m^3^ increment of PM_10_ among all participants, participants with AO and participants without AO, assuming the PM_10_ obtained by the air quality modelling system (WRF-CAMx).

|  | **% Change per 1 µg/m^3^ of PM_10_ increment** | | | | |
| --- | --- | --- | --- | --- | --- |
|  | TG | TC | HDL-C | LDL-C | HbA1C |
| **All included participants(n=2390)** | | | | | |
| Not adjusted model | -0.60  (-1.25; 0.05) | -0.02  (-0.43; 0.38) | 0.06  (-0.33; 0.46) | -0.31  (-1.06; 0.45) | -0.07  (-0.27; 0.14) |
| *Adjusted model | 0.14  (-0.89; 1.19) | 0.04  (-0.42; 0.50) | 0.02  (-0.44; 0.47) | -0.20  (-1.02; 0.62) | -0.03  (-0.25; 0.20) |
| **Participants with AO (n=1831)^a^** |  | | | | |
| Not adjusted model | -0.03  (-0.77; 0.72) | 0.06  (-0.37; 0.48) | -0.10  (-0.55; 0.36) | -0.14  (-0.91; 0.64) | -0.03  (-0.30; 0.24) |
| *Adjusted model | 0.43  (-0.67; 1.55) | 0.09  (-0.37; 0.54) | -0.08  (-0.66; 0.51) | -0.09  (-0.90; 0.74) | -0.03  (-0.33; 0.27) |
| **Participants without AO (n=536)** |  | | | | |
| Not adjusted model | -1.25  (-2.75; 0.28) | -0.11  (-0.62; 0.40) | -0.10  (-0.76; 0.57) | -0.47  (-1.48; 0.55) | 0.17  (-0.05; 0.38) |
| *Adjusted model | -0.63  (-2.02; 0.78) | 0.01  (-0.61; 0.63) | -1.36  (-0.51; 0.51) | -0.36  (-1.54; 0.83) | 0.14  (-0.05; 0.33) |

*adjusted for age, sex, educational level, occupation, smoking status, excessive alcohol consumption, unhealthy diet, sedentary. Individual allocated 1-year average temperature was not included because PM_10_ obtained by air quality modelling already taking into account meteorological variables including temperature.

^a^ Participants without available data on waist or height measurements (n=17) and consequently without AO data were excluded from the stratified analysis.

Abbreviations: AO- Abdominal Obesity; TC - Total Cholesterol; HDL-C – High density Lipoprotein cholesterol; LDL-C – Low density lipoprotein cholesterol; TG – Triglycerides.; PM- Particulate matter.

**Table S4-** Percent changes in TG, TC, HDL-C, LDL-C and HbA1C per 1 µg/m^3^ increment of PM_10_ among all participants, participants with AO and participants without AO after exclusion of participants with diagnosed dyslipidaemia or taking lipid-lowering medication (in the TG, CT, HDL-C and LDL-C modelling) and diabetic participants or taking medication for diabetes treatment (in the HbA1C modelling).

|  | **% Change per 1 µg/m^3^ of PM_10_ increment** | | | | |
| --- | --- | --- | --- | --- | --- |
|  | TG | TC | HDL-C | LDL-C | HbA1C |
| **All included participants**  **(n=1709, n=2165 for HbA1C)** | | | | | |
| Not adjusted model | -0.03  (-1.65; 1.629 | 0.09  (-0.70; 0.89) | 0.30  (-0.33; 0.94) | -0.95  (-2.03; 0.15) | -0.05  (-0.28; 0.18) |
| *Adjusted model | **2.08**  **(0.40; 3.80)** | 0.63  (-0.22; 1.48) | -0.11  (-0.74; 0.52) | 0.50  (-0.29; 1.30) | 0.01  (-0.33; 0.35) |
| **Participants with AO ^a^**  **(n=1208, n=1625 for HbA1C)** | | | | | |
| Not adjusted model | 0.93  (-1.02; 2.92) | 0.21  (-0.60; 2.92) | 0.00  (-0.62; 0.63) | -0.64  (-1.71; 0.43) | -0.07  (-0.38; 0.25) |
| *Adjusted model | **2.30**  **(0.22; 4.43)** | 0.66  (-0.18; 1.50) | -0.28  (-1.06; 0.50) | 0.56  (-0.25; 1.38) | -0.01  (-0.44; 0.42) |
| **Participants without AO**  **(n=482, n=519 for HbA1C)** | | | | | |
| Not adjusted model | -1.73  (-3.63; 0.20) | 0.10  (-0.74; 0.94) | 0.47  (-0.54; 1.49) | -1.20  (-2.48; 0.09) | 0.22  (-0.04; 0.49) |
| *Adjusted model | 1.08  (-1.41; 3.65) | 0.74  (-0.30; 1.79) | 0.40  (-0.59; 1.40) | 0.38  (-0.69; 1.47) | 0.10  (-0.28; 0.48) |

*adjusted for age, sex, educational level, occupation, smoking status, excessive alcohol consumption, unhealthy diet, sedentary and individual allocated 1-year average temperature.

^a^ Participants without available data on waist or height measurements (n=19, n=21 for the HbA1C) and consequently without AO data were excluded from the stratified analysis.

Abbreviations: AO- Abdominal Obesity; TC - Total Cholesterol; HDL-C – High density Lipoprotein cholesterol; LDL-C – Low density lipoprotein cholesterol; TG – Triglycerides.; PM- Particulate matter.

**Table S5-** Percent changes in TG, TC, HDL-C, LDL-C and HbA1C per 1 µg/m^3^ increment of PM_10_ among all participants, participants with AO and participants without AO, considering waist-to-hip ratio as the measure of Abdominal Obesity.

|  | **% Change per 1 µg/m^3^ of PM_10_ increment** | | | | |
| --- | --- | --- | --- | --- | --- |
|  | TRIG | TC | HDL-C | LDL-C | HbA1C |
| **All included participants (n=2390)** | | | | | |
| Not adjusted model | 0.19  (-1.08; 1.48) | 0.09  (-0.52; 0.70) | 0.12  (-0.43; 0.68) | -0.89  (-1.87; 0.11) | -0.09  (-0.37; 0.19) |
| *Adjusted model | **1.70**  **(0.11; 3.32)** | 0.59  (-0.07; 1.24) | -0.20  (-0.74; 0.33) | 0.47  (-0.20; 1.15) | -0.01  (-0.48; 0.47) |
| **Participants with AO (n=1583)^a^** |  | | | | |
| Not adjusted model | 0.84  (-0.40; 2.09) | 0.11  (-0.52; 0.74) | -0.28  (-0.77; 0.21) | -0.67  (-1.66; 0.33) | -0.11  (-0.52; 0.30) |
| *Adjusted model | **1.68**  **(0.03; 3.35)** | 0.50  (-0.20; 1.21) | -0.46  (-1.16; 0.24) | 0.41  (-0.34; 1.16) | -0.07  (-0.75; 0.61) |
| **Participants without AO (n=791)** |  | | | | |
| Not adjusted model | -1.06  (-3.24; 1.18) | 0.15  (-0.29; 0.58) | 0.62  (-0.34; 1.5) | **-1.19**  **(-2.10; -0.27)** | **0.17**  **(0.03; 0.32)** |
| *Adjusted model | 1.35  (-0.51; 3.25) | 0.78  (-0.07; 1.62) | 0.51  (-0.46; 1.35) | 0.54  (-0.20; 1.28) | 0.11  (-0.12; 0.34) |

*adjusted for age, sex, educational level, occupation, smoking status, excessive alcohol consumption, unhealthy diet, sedentary and individual allocated 1-year average temperature.

^a^ Participants without available data on waist or hip measurements (n=16) and consequently without AO data were excluded from the stratified analysis.

Abbreviations: AO- Abdominal Obesity; TC - Total Cholesterol; HDL-C – High density Lipoprotein cholesterol; LDL-C – Low density lipoprotein cholesterol; TG – Triglycerides.; PM- Particulate matter.

**Figure S1-** A Directed Acyclic Graph (DAG) for the association between long-term air pollution exposure (PM10 annual mean values) and blood lipid and glucose levels. The dashed row representing the modification effect of abdominal obesity was not included in the analysis of the minimal sufficient adjustment set of variables. Abbreviations: HbA1c, Glycated haemoglobin; TC, Total Cholesterol; HDL-C, High-Density Lipoprotein Cholesterol; LDL-C, Low-Density Lipoprotein Cholesterol; TG, Triglycerides; PM, Particulate matter; WHtR, Waist-to-Height Ratio.
